# CircCRIM1 Ameliorates Endothelial Cell Angiogenesis in Aging through the miR-455-3p/Twist1/VEGFR2 Signaling Axis

**DOI:** 10.1155/2022/2062885

**Published:** 2022-10-08

**Authors:** Lei Zhao, Rencong Chen, Jiacong Qiu, Yingxiong Huang, Chong Lian, Xiaonan Zhu, Jin Cui, Siwen Wang, Shenming Wang, Zuojun Hu, Jinsong Wang

**Affiliations:** ^1^Department of Vascular Surgery, The First Affiliated Hospital of Sun Yat-sen University, NO. 58, Zhong Shan Er Lu, Guangzhou 510080, China; ^2^National-Guangdong Joint Engineering Laboratory for Diagnosis and Treatment of Vascular Diseases, NO. 58, Zhong Shan Er Lu, Guangzhou 510080, China; ^3^Guangdong Engineering and Technology Center for Diagnosis and Treatment of Vascular Diseases, NO. 58, Zhong Shan Er Lu, Guangzhou 510080, China; ^4^Department of vascular Surgery, The Second Affiliated Hospital of Nanchang University, Nanchang University, Nanchang, Jiangxi 330006, China; ^5^Department of Emergency, The First Affiliated Hospital of Sun Yat-sen University, NO. 58, Zhong Shan Er Lu, Guangzhou 510080, China; ^6^Department of Endovascular Surgery, The First Affiliated Hospital of Zhengzhou University, 450000 Zhengzhou, Henan, China; ^7^Department of Pharmacology Laboratory, Zhongshan School of Medicine, Sun Yat-sen University, NO. 58, Zhong Shan Er Lu, Guangzhou 510080, China; ^8^Division of Vascular and Plastic Surgery, Guangdong Provincial People's Hospital, Guangdong Academy of Medical Sciences, Guangzhou 510080, China

## Abstract

**Background:**

Aging leads to vascular endothelial cell senescence. Decreased expression of VEGFA and VEGFR2 plays a crucial role in impairing angiogenesis in senescent endothelial cells. Noncoding RNAs, including circular RNAs (circRNAs) and microRNAs (miRNAs), regulate endothelial cell proliferation, differentiation, apoptosis, and migration and participate in the occurrence and development of vascular diseases. However, the mechanism of noncoding RNAs in age-related vascular endothelial dysfunction remains unclear. Here, we aimed to identify the circRNA that is associated with VEGF/VEGFR2 signaling pathway activation in angiogenesis.

**Methods:**

Immunoblotting, quantitative reverse transcription-polymerase chain reaction (qRT–PCR), *in vitro* and *in vivo* experiments, luciferase assays, and chromatin immunoprecipitation followed by qRT–PCR (ChIP–qPCR) assays were performed to clarify the roles played by circCRIM1 in mouse aortic endothelial cell (MAEC) angiogenesis.

**Results:**

CircCRIM1 expression was downregulated in both an aging mouse model of lower limb ischemia *in vivo* and aging MAECs *in vitro*. Overexpressing circCRIM1 mediated through a plasmid or adeno-associated virus (AAV) reversed the downregulation of angiogenesis-related phenotype acquisition during aging. MiR-455-3p was confirmed to be a potential target of circCRIM1 through luciferase assays followed by RNA fluorescence in situ hybridization (FISH), which revealed the colocalization of circCRIM1 and miR-455-3p. CircCRIM1 was found to be a competitive endogenous RNA that sponged miR-455-3p and regulated angiogenesis-related phenotypes in MAECs. Furthermore, Twist1 was found to be downstream of miR-455-3p. A ChIP–qPCR assay showed that Twist1 promoted VEGFR2 expression by binding to the promoter region, playing a vital role in angiogenesis.

**Conclusions:**

Decreased expression of circCRIM1 impaired angiogenesis in aging via the miR-455-3p/Twist1/VEGFR2 axis. Our findings suggest that overexpression of circCRIM1 may be an effective therapeutic strategy for promoting ischemic lower limb blood flow recovery.

## 1. Introduction

Peripheral artery disease (PAD) is a severe medical challenge that significantly impacts people's lives and health in aging societies worldwide. In China, 6.6% of adults more than 35 years old (approximately 45.3 million people) have PAD, and the prevalence of PAD among people 60 years old and older ranges from 2.8% to 15.3% [1]. Age is an independent factor affecting vascular homeostasis, leading to impaired angiogenesis [2]. Endothelial cells (ECs), which bridge blood and vascular tissue to deliver nutrients and active molecules, play important roles in maintaining vascular homeostasis, including in vascular growth and differentiation, as well as vascular tone regulation [3]. Therefore, elucidation of the biological role and molecular mechanism of ECs in aging for early PAD detection and therapeutic target development is of high clinical significance.

Recently, noncoding RNAs that play key roles in regulating gene expression have attracted considerable attention. As a type of noncoding RNA, circular RNAs (circRNAs) tend to form a ring structure by covalent bonding in the absence of a 5′-end cap and a 3′-end poly (A) tail [4]. Benefiting from their stability, conservation, endogenous nature, and abundance, circRNAs have been associated with an increasing number of functions in epigenetic regulation in various organisms [5]. Studies have shown that circRNAs are of considerable importance to regulate epigenetic modifications that affect vascular EC function, including proliferation, differentiation, migration, and tube formation, thus mediating the occurrence and development of various vascular diseases [6, 7]. However, the effects of circRNAs on vascular EC angiogenesis in aging and the related mechanism remain unclear. Consequently, there is an urgent need for research on differentially expressed circRNAs in young and old ECs to explore the mechanisms of age-related angiogenesis, especially to provide new insights into early diagnostic markers and therapeutic targets for PAD.

In our study, we found that the circular RNA mmu_circ_0007020, a putative circRNA originating from the cysteine-rich transmembrane BMP regulator 1 (chordin like) (CRIM1) mRNA, was significantly downregulated in vascular endothelial cells in elderly mice by performing a whole-transcriptome sequencing data screening. To explore the underlying functional role played by mmu_circ_0007020 (circCRIM1) in ECs, *in vitro* and *in vivo* experiments were performed and showed that circCRIM1 sponged miR-455-3p and thus enhanced endothelial angiogenesis in aging, targeting Twist1/VEGFR2 signaling axis.

## 2. Materials and Methods

### 2.1. Clinical Samples and Ethical Statements

This study has been approved by the ethics committee of the First Affiliated Hospital of Sun Yat-sen University (Approval No. [2022]337) and is in accordance with the Helsinki Declaration. The arterial tissues were derived from patients over 65 years old that were performed abdominal aortic aneurysmectomy with artery reconstruction, or patients less than 30 years who volunteer to donate their bodies with informed consent. Samples were sectioned from the distal iliac arterial tissues adjacent to the lesions in old patients, or the normal iliac arteries in young patients.

### 2.2. Cell Culture

MAECs were isolated from C57BL/6 mice and were cultured as described in our previous study [8]. Briefly, aortas were dissected from euthanized mice and were digested by trypsin. The harvested MAECs were cultured in DMEM (Thermo Fisher Scientific) medium containing 10% fetal bovine serum (FBS, Thermo Fisher Scientific), and 1% penicillin/streptomycin in an incubator at 37°C with 5% CO_2_. The old mice were 18 months old, and the young mice were 4 weeks old [9]. MAECs were extracted from the same sample batch obtained from 4 mice, and the experiments were performed at passages 3 to 5. The mouse studies and experimental procedures were approved by the Ethics Committee for Experimental Animals of the First Affiliated Hospital of Sun Yat-sen University (Approval No. [2021]581).

### 2.3. Cell Transfection

CircCRIM1 short interfering RNA (siRNA, RiboBio) and plasmid (Geneseed) were incubated with Lipofectamine 3000 (Thermo Fisher Scientific) in a reduced serum medium and then used for cell transfection with the concentration of 50 nmol and 2.5 *μ*g/mL, respectively. Transfected cells were cultured at 37°C in an incubator with 5% CO_2_. RNA and total protein were extracted from 48 to 72 h after transfection. Transfected cells were performed in subsequent experiments after 48 h transfection. All siRNA sequences used for transfection are listed in Supplementary Material Table I.

### 2.4. RNA Extraction and qRT–PCR

TRIzol reagent (Thermo Fisher Scientific) was used to extract total RNA from MAECs in the old group and young group. And then the RNAs were performed on reverse transcription into cDNA using a first-strand cDNA synthesis kit (TaKaRa) after purity and spectrophotometric identification of concentration. SYBR green PCR Master Mix (TaKaRa) was used for subsequent qRT–PCR detected in a LightCycler 480 system (Roche). GAPDH expression was compared with circCRIM1 and Twist1 expression levels as the internal reference gene, and U6 expression was compared with microRNA (miRNA) expression as the internal reference gene. All primer sequences are listed in Supplementary Material Table II.

### 2.5. RNase R Experiment

RNAs from MAECs (10 *μ*g) were incubated with ribonuclease R (RNase R, 3 U/*μ*g, Geneseed) at 37°C for 20 min. Then the treated RNAs were reversely transcribed using different primers and then detected by qRT–PCR.

### 2.6. RNA Immunoprecipitation

RNA immunoprecipitation kit (Geneseed) was used for RNA immunoprecipitation (RIP) analysis. First, MAECs were transfected by a plasmid or vector, respectively. And then RNAs extracted from each group were incubated with magnetic beads bound to anti-Argonaute 2 (AGO2) or anti-IgG antibody (Millipore) at 4°C for 6 h. Then the magnetic beads were cleaned and purified with columns to eliminate the DNAs and RNAs. Finally, proteins were isolated by acetone and ethyl alcohol and subsequently determined by western blotting.

### 2.7. ChIP–qPCR

SimpleChIP® Enzymatic Chromatin IP Kit (Agarose Beads) (Cell Signaling Technology, Inc.) was used for the ChIP experiment. Proteins and nucleic acids in MAECs were cross-linked by treatment with a 1% formaldehyde solution. Then, MAECs were incubated with the protease inhibitor cocktail at 4°C and were collected for subsequent experiments. The cross-linked chromatin was extracted from MAECs by lysis buffer, followed by purification procedures and sonication. After being treated with RNase A and Proteinase K as required, the qualified chromatin sample was incubated at 4°C overnight with 1 × ChIP buffer supplemented with Twist1 antibody (Santa Cruz Biotechnology, Inc.). On the next day, the Protein G Agarose Beads were used for further immunoprecipitation and purification. Finally, the pellets were eluted and reverse cross-linked to release ChIP DNA, and then the DNAs were prepared and used for subsequent qRT–PCR. All protocol details were available in the manual. The primers for VEGFR2 used in ChIP–qPCR are listed in Supplementary Material Table III.

### 2.8. Nucleic Acid Electrophoresis

qRT–PCR products of cDNA and gDNA were performed on electrophoresis at 100 V for 45 minutes in 3% agarose gel supplemented with TAE buffer. Nucleic acid fragments of 100-500 bp were identified by using a DNA marker (Accurate Biology). Then the gel was irradiated and imaged by an ultraviolet (UV) ray detector.

### 2.9. Fluorescence *In Situ* Hybridization (FISH)

Cy3-labeled circCRIM1 probe and FITC-labeled miR-455-3p probe were synthesized by GenePharma (Shanghai, China). FISH was carried out to determine the localization of circCRIM1 and miR-455-3p in MAECs. CircCRIM1 and miR-455-3p probes were hybridized and incubated overnight. The Zeiss LSM710 confocal laser scanning microscope (Zeiss Instruments) captured images, and the Otsu FLTER software automatically sets thresholds to quantify the immunofluorescence signals of circCRIM1 and miR-455-3p. The colocated signals (Pearson Manders' coefficients) were calculated based on representative cell images by the JACoP (i.e., co-location plug-in). The probe sequences of circCRIM1 and miR-455-3p refer to Supplementary Material Table IV.

### 2.10. Western Blotting

Frozen RIPA buffer (Thermo Fisher Scientific) containing protease and phosphatase inhibitors was used for lysis. After using a BCA Protein Analysis Kit (Thermo Fisher Scientific) to detect the concentration, and equivalent quantities (30 *μ*g) of protein samples were loaded onto 10% gels for SDS-PAGE, followed by transference to PVDF membrane (Millipore). Then, the membrane was blocked with TBST containing 5% bovine serum albumin (BSA, BioFroxx), and rabbit primary antibody against Twist1 was applied to incubate at 4°C overnight, while GAPDH was used as control. And then, the secondary antibody was applied to membrane incubation for an hour at room temperature. NovexECL (Invitrogen) visualized the expression of proteins and the signal quantification was analyzed by ImageJ software (version 1.8.0).

### 2.11. Luciferase Assay

CircCRIM1 sequences with miR-455-3p binding site mutations were acquired with a QuikChange II Locus-Directed Mutation Kit (Stratagene). Wild-type-(Wt-) miR-455-3p and mutant (MUT)-miR-455-3p purchased from Geneseed were cotransfected with the negative control (NC) or circCRIM1 sequence into HEK-293 T cells by Lipofectamine 3000 (Thermo Fisher Scientific). After 24 h transfection, luciferase activities were detected using a dual-luciferase reporting and detection kit (TransGen Biotech).

### 2.12. *In Vitro* Wound Healing Assay

Treated MAECs were inoculated in a 6-well culture plate until the confluence of 90% was reached. The cell monolayer was scratched to create a reference point by a 1 ml sterile pipette tip, and then they were washed with sterile phosphate-buffered saline (PBS) buffer. Reference images before the experiment were recorded by a microscope (Olympus Co., Ltd.). After being cultured in the serum-free medium for one day, the cell images of the same area were obtained by microscopy again, and statistical analysis of the scratch healing area was performed with ImageJ software.

### 2.13. Transwell Assay

A chamber with an 8 *μ*m aperture (Corning Life Sciences) was placed into a twenty-four-well plate with the lower chamber containing 400-600 *μ*L of complete medium supplemented with 15% FBS, in which the upper chamber was inoculated with treated MAECs that were suspended by 300 *μ*L of serum-free medium. After 24 h of culture, the cells were fixed with 4% paraformaldehyde for 20 min and were subsequently stained with crystal violet for 15 min. Finally, the nonmigrating cells were removed from the inner membrane by gently wiping the upper chamber wall with cotton balls, and the migrating cells in 5 random areas were counted through light microscopy (Olympus Co., Ltd.).

### 2.14. Tube Formation Experiment

Twenty-four-well plates were precooled overnight at 4°C, and each well was padded with 200 *μ*L of matrix glue (Corning Life Sciences), placed in a cold closet for 10 min to level the matrix glue, followed by culture in a cell incubator for half an hour to enable matrix coagulation. MAECs with a density of 150 thousand/well were added into the solidified matrix gel and cultured for six hours. At last, a light microscope (Olympus Co., Ltd.) was used to take images. The tube length measurements were performed by the ImageJ software.

### 2.15. 5-Ethynyl-2′-Deoxyuridine (EdU) Cell Proliferation Assay

MAECs were cultured in nighty-six-well plates until 60-70% confluence was achieved. The proliferative capacity of MAECs was assessed according to the instructions of a C10310-1/-2/-3 kit (RiboBio), and cell proliferation capacity was assessed by measuring the proportion of EdU-positive cells with a fluorescence microscope (Leica).

### 2.16. Animal Model

All procedures were approved by the Animal Ethics Committee of the First Affiliated Hospital of Sun Yat-sen University (Approval No. [2021]581). C57BL/6 mice aged eighteen months or four weeks (GemPharmatech Co., Ltd.) was used to establish an animal model of unilateral hindlimb ischemia. The mice were anesthetized with isoflurane and were conducted by a 1-cm incision in the right inguinal region. After subcutaneous tissue separation followed by the femoral artery sheath open, the femoral artery was separated from the concomitant vein and nerve. Then, the femoral arteries were ligated with 7-0 silk thread at both ends and subsequently cut off in between. Finally, the incision was sutured. The other side of the left hindlimb was used as the control. The mice were randomly divided into 4 groups, including old mice injected with adeno-associated virus serotype 9 (AAV9)-circCRIM1, old mice injected with AAV9-NC, old mice injected with PBS, and young mice injected with PBS. All AAVs were designed and obtained from GeneChem (Shanghai, China). To develop overexpression AAV, circCRIM1 was subcloned into a GV526 AAV9 vector (CAG-MCS-SV40 PolyA). AAV9 (30 *μ*L in each leg for 10^10^ VG of virus volume) was injected and PBS (30 *μ*L in each leg) was injected into the calf gastrocnemius muscle two weeks before the operation, and the left hindlimb on the other side as the control. Blood flow was observed with an animal blood flow imaging apparatus before and on days 0, 3, 7, 14, 21, and 28 after surgery. The mice were euthanatized via intraperitoneal injection of high-dose pentobarbital on day 28 after the operation, and the gastrocnemius muscles were analyzed by immunohistochemistry, qRT–PCR, and other experiments.

### 2.17. Blood Flow Measurements

An animal blood flow imaging apparatus, PeriCamPS I (Perimed), was used to measure lower limb blood flow in mice 0, 3, 7, 14, 21, and 28 days after surgery in an environment with suitable temperature and humidity, without excessive light or noise. PIMSoft (Perimed) software was used to analyze the blood flow recovery index by calculating the perfusion ratio of ischemic limbs to normal limbs.

### 2.18. Immunohistochemistry

Hydrogen peroxide blocker-treated mouse muscle sections (3 *μ*m) were incubated with anti-CD31 and anti-Twist1 primary antibodies overnight. Then, the sections were washed and subsequently incubated with secondary antibodies, followed by 3, 3′-diaminobenzidine reagent (DAB; Solarbio) treatment and hematoxylin staining. Olympus BX51 microscope (Olympus) was used to take images of the muscle sections.

### 2.19. Statistical Analysis

All data are presented as the mean ± standard deviation (SD) and were analyzed by the GraphPad Prism 8.0.1. Comparisons between two groups were analyzed by independent sample *T*-test, and a one-way analysis of variance (ANOVA) was performed for comparisons between three or more groups. *P* value < 0.05 was considered statistically significant.

## 3. Results

### 3.1. CircCRIM1 characteristics

**Figure 1:**
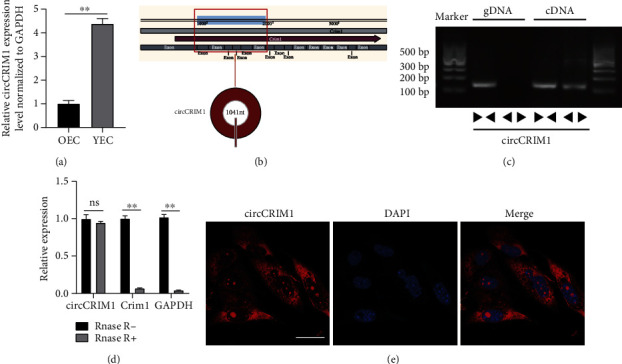
Profiling of circCRIM1. (a) Comparison of the circCRIM1 expression in OECs and YECs, normalized to GAPDH (*n* = 3). (b) Information of circCRIM1 from the circBase database. (c) Agarose gel electrophoresis results showed that divergent circCRIM1 primers were amplified by cDNA but not by gDNA. (d) With or without RNase R, comparison of the circCRIM1 and linear CRIM1 mRNA expression in MAECs. (e) FISH assay showed that circCRIM1 was predominantly localized in the cytoplasm. (Magnification, 400×, scale bar, 15 *μ*m). (The data are expressed as the mean ± SD, ^∗∗^*P* < 0.01 versus the negative control, ns means no significance).

We found that circCRIM1 expression in ECs of elderly mice was downregulated compared with that of young mice based on our previous whole-transcriptome sequencing [8]. To determine the expression of circCRIM1 in aging, we investigated the expression of circCRIM1 in 5th generation old endothelial cells (OEC) and young endothelial cells (YEC) using qRT–PCR. The results showed that circCRIM1 expression was significantly lower in the OEC than in YEC (Figure 1(a)). Information on circCRIM1 from the circBase database showed that in mice circCRIM1 is derived from the CRIM1 gene on chromosome 17 (Figure 1(b)). The code in circBase is mmu_circ_0007020 and shows 1041 bases. Agarose gel electrophoresis revealed that divergent circCRIM1 primers were amplified by cDNA but not by gDNA (Figure 1(c)). CircCRIM1 was resistant to RNase R digestion, while CRIM1 mRNA and GAPDH were not amplified by cDNA (Figure 1(d)). To verify the subcellular localization of circCRIM1, an RNA FISH assay was implemented and showed that circCRIM1 was predominantly localized in the cytoplasm (Figure 1(e)).

### 3.2. CircCRIM1 Promotes MAECs' Proliferative, Migratory, and Tube Formative Capacities In Vitro

**Figure 2:**
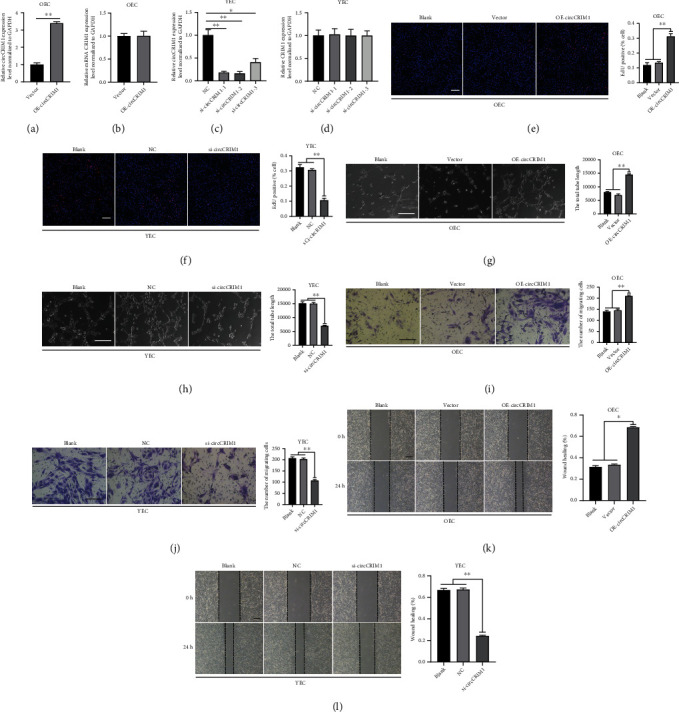
CircCRIM1 promotes MAECs' proliferative, migratory, and tube formative capacities *in vitro*. (a) CircCRIM1 expression was upregulated by transfecting OE-circCRIM1 into OEC (*n* = 3). (b) The CRIM1 mRNA expression was not altered significantly after circCRIM1 overexpression (*n* = 3). (c) CircCRIM1 expression was knockdown by transfecting circCRIM1 siRNAs into YEC (*n* = 3). (d) The CRIM1 mRNA expression was not altered significantly after transfection of circCRIM1 siRNAs (*n* = 3). (e) Overexpressing circCRIM1 increased OEC proliferation capacity in the EdU assay (*n* = 3, scale bar, 100 *μ*m). (f) CircCRIM1 siRNAs diminished YEC proliferation in the EdU assay (*n* = 3, scale bar, 100 *μ*m). (g) Overexpressing circCRIM1 promoted OEC tube formation (*n* = 3, scale bar, 100 *μ*m). (h) CircCRIM1 siRNAs impaired YEC tube formation (*n* = 3, scale bar, 100 *μ*m). (i) Overexpressing circCRIM1 ameliorated OEC migration in the Transwell assay (*n* = 3, scale bar, 100 *μ*m). (j) CircCRIM1 siRNAs decreased YEC migration in the Transwell assay (*n* = 3, scale bar, 100 *μ*m). (k) Overexpressing circCRIM1 invigorated OEC migration in the *in vitro* wound healing assay (*n* = 3, scale bar, 100 *μ*m). (l) CircCRIM1 siRNAs diminished YEC migration in the *in vitro* wound healing assay (*n* = 3, scale bar, 100 *μ*m). (The data are presented as the *mean* ± *SD*, ^∗^*P* < 0.05, ^∗∗^*P* < 0.01 versus the negative control).

To clarify whether circCRIM1 plays a role in MAEC angiogenesis, we designed an overexpression vector of circCRIM1 (OE-circCRIM1) that was subsequently transfected into OEC. qRT–PCR showed that circCRIM1 in the OEC was successfully overexpressed (Figure 2(a)) and that the expression of CRIM1 mRNA was not altered (Figure 2(b)).

To silence endogenous circCRIM1 expression, we transfected circCRIM1 siRNA into YEC, and siRNA-NC was transfected into YEC to establish the control. qRT–PCR showed that siRNAs significantly knocked down the expression of circCRIM1 in YEC and that si-circCRIM1-2 showed the most obvious effect, and therefore, si-circCRIM1-2 was selected for use in subsequent experiments (Figure 2(c)). Notably, none of the siRNAs altered the expression of CRIM1 mRNA (Figure 2(d)).

A series of functional experiments were conducted to further examine the function of circCRIM1 in angiogenesis-related phenotype acquisition. After circCRIM1 expression was upregulated by transfecting OE-circCRIM1 into OEC, MAEC showed increased proliferation capacity, as evaluated by EdU assay (Figure 2(e)), while circCRIM1 siRNAs diminished MAEC proliferation among the YEC populations (Figure 2(f)). Furthermore, CircCRIM1 increased the OEC tube formation number (Figure 2(g)), and tube formation was impaired in the YEC transfected with circCRIM1 siRNA (Figure 2(h)). As shown in Figures 2(i) and 2(j), the Transwell assay results showed that upregulation of circCRIM1 expression ameliorated OEC migration, while silencing of circCRIM1 expression resulted in a low YEC migration rate. Similarly, *in vitro* wound healing assay results supported the finding suggesting that overexpressing circCRIM1 invigorated OEC migration (Figure 2(k)), and transfection of circCRIM1 siRNA took the edge off the migratory capacity of the YEC (Figure 2(l)).

### 3.3. MiR-455-3p is the Downstream Target of CircCRIM1

**Figure 3:**
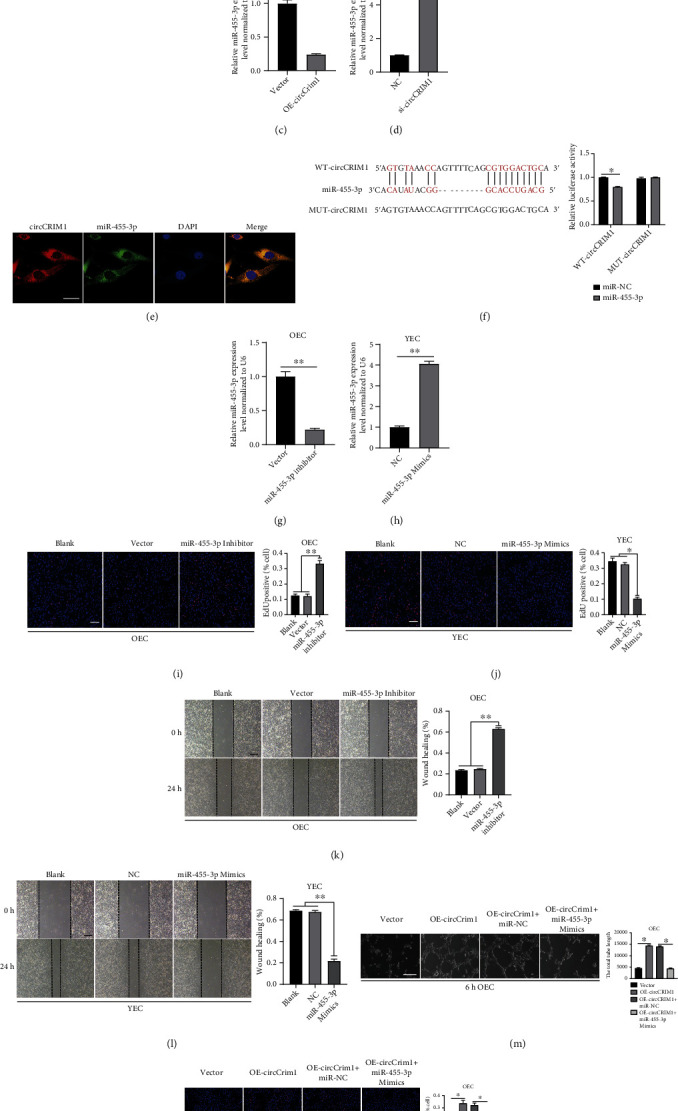
MiR-455-3p is the downstream target of circCRIM1 and inhibits the proliferation, and migration of MAECs. (a) RIP assay was performed in OEC, followed by western blot to detect AGO2 expression. (b) MiR-455-3p was significantly overexpressed in the OEC compared with the YEC (*n* = 3). (c) MiR-455-3p expression was downregulated by overexpressing circCRIM1 in OEC (*n* = 3). (d) MiR-455-3p expression was upregulated in YEC transfected by circCRIM1 siRNA (*n* = 3). (e) The results of the FISH experiments showed the colocalization of circCRIM1 and miR-455-3p (magnification, 400×, scale bar, 15 *μ*m) (*n* = 3, analyzed 8 cells). (f) Schematic diagrams of the circCRIM1-WT and circCRIM1-MUT structure. Luciferase assay proved that miR-455-3p suppressed the luciferase activity of the circCRIM1 3′-UTR-WT construct but not the circCRIM1 3′-UTR-MUT construct. (g) qRT–PCR results showed successful miR-455-3p silence in OEC (*n* = 3). (h) qRT–PCR results showed successful miR-455-3p upregulation in YEC (*n* = 3). (i) OEC proliferation capacity was increased by the inhibitor of miR-455-3p (*n* = 3, scale bar, 100 *μ*m). (j) YEC proliferation capacity was diminished by the miR-455-3p mimic (*n* = 3, scale bar, 100 *μ*m). (k) Transfection of the inhibitor of miR-455-3p invigorated OEC migration in the *in vitro* wound healing assay (*n* = 3). (l) Transfection of the miR-455-3p mimic impaired YEC migration in the *in vitro* wound healing assay (*n* = 3). (m) Tube formation assay of OECs treated with vector, OE-circCRIM1, OE-circCRIM1+miR-NC, or OE-circCRIM1+miR-455-3p mimics (*n* = 3, scale bar, 100 *μ*m). (n) EdU assay of OECs treated with vector, OE-circCRIM1, OE-circCRIM1+miR-NC or OE-circCRIM1+miR-455-3p mimics (*n* = 3, scale bar, 100 *μ*m). (The data are presented as the mean ± SD, ^∗^*P* < 0.05, ^∗∗^*P* < 0.01 versus the negative control).

As circCRIM1 is abundantly expressed in the cytoplasm, we investigated how it regulates angiogenesis in MAECs. RIP experiments with OEC showed that circCRIM1 interacted with the AGO2 protein, which bound circRNA and miRNA to fulfill the competing endogenous RNA (ceRNA) function (Figure 3(a)). To identify the miRNAs that were potentially targeted by circCRIM1, we performed bioinformatics analysis using the miRanda database and RNAhybrid database. Among the potential miRNAs, we verified that only miR-455-3p was significantly overexpressed in the OEC compared with YEC (Figure 3(b)). Then, qRT–PCR was performed to verify that circCRIM1 overexpression resulted in the downregulation of miR-455-3p in the OEC (Figure 3(c)). In contrast, miR-455-3p was found to be upregulated after circCRIM1 knockdown in YECs (Figure 3(d)). The results of the FISH experiments showed the colocalization of circCRIM1 and miR-455-3p (Figure 3(e)). To further validate the potential binding sites of circCRIM1 and miR-455-3p, a luciferase assay was performed to prove that miR-455-3p suppressed the luciferase activity of the circCRIM1 3′-UTR-WT construct but not the circCRIM1 3′-UTR-MUT construct (Figure 3(f)). Our results indicated that the specific interaction between circCRIM1 and miR-455-3p exists.

### 3.4. MiR-455-3p Inhibits MAECs Proliferation and Migration

To further determine the functional role played by miR-455-3p in MAECs, we transfected an inhibitor of miR-455-3p into OEC to silence miR-455-3p and simultaneously overexpressed miR-455-3p in YEC through transfection of mimic. qRT–PCR was performed to prove the transfection efficacy (Figures 3(g) and 3(h)). As shown in Figures 3(i) and 3(j), proliferation was significantly increased by the miR-455-3p inhibitor transfected into OEC but impaired by transfection of the miR-455-3p mimic in YEC, as determined by the EdU assay. The *in vitro* wound healing assay showed an enhanced migratory capacity of the OEC because of miR-455-3p silencing (Figure 3(k)), while upregulation of miR-455-3p resulted in a weakened migratory capacity of YEC (Figure 3(l)).

### 3.5. CircCRIM1 Promotes Angiogenesis by Sponging MiR-455-3p in MAECs

Furthermore, we performed rescue experiments to determine whether circCRIM1 regulates angiogenesis by sponging miR-455-3p and to discern its function. The OEC tube formation rate was accelerated because of circCRIM1 upregulation, but this augmentation was reduced by cotransfection with the miR-455-3p mimic (Figure 3(m)). Moreover, overexpressing circCRIM1 increased the OEC proliferation, but failed to maintain this increase in proliferation in the presence of the miR-455-3p mimic (Figure 3(n)). These results suggested that miR-455-3p is a downstream target of circCRIM1, playing a vital role in regulating MAEC angiogenesis.

### 3.6. Twist1 is the Downstream Target of MiR-455-3p

**Figure 4:**
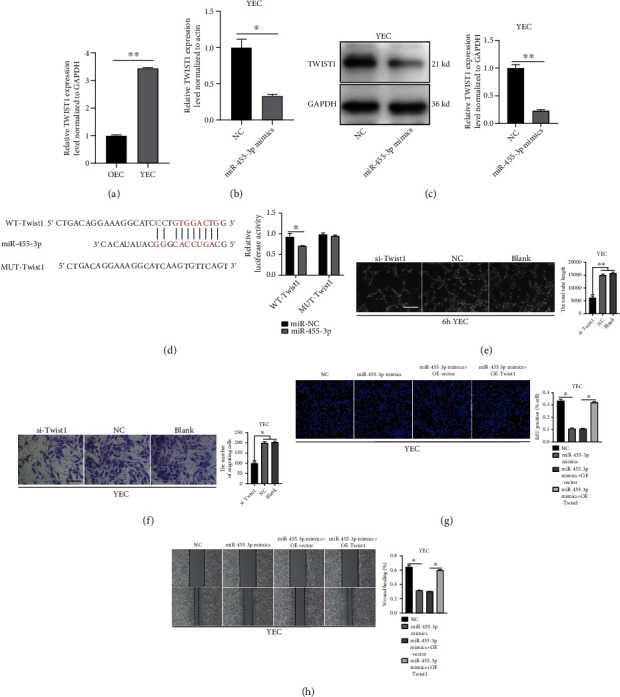
Twist1 is the downstream target of miR-455-3p. (a) qRT–PCR results showed that the Twist1 mRNA level was significantly lower in OEC than in YEC (*n* = 3). (b) The Twist1 mRNA level was decreased in YEC transfected with miR-455-3p mimic (*n* = 3). (c). Western blot analysis showed that the miR-455-3p mimic resulted in decreased Twist1 expression. (d) Schematic diagrams of the Twist1-WT and Twist1-MUT structure. Luciferase assay proved that Twist1-WT 3′UTR but not the MUT 3′UTR was suppressed by miR-455-3p mimics. (e) Silencing Twist1 diminished YEC tube formation (*n* = 3, scale bar, 100 *μ*m). (f) Silencing Twist1 diminished YEC migratory capacity in the Transwell assay (*n* = 3, scale bar, 100 *μ*m). (g) EdU assay of YEC treated with NC, miR-455-3p mimics, miR-455-3p mimics +vector or miR-455-3p mimics +OE-Twist1 (*n* = 3). (h) *In vitro* wound healing assay of YEC treated with NC, miR-455-3p mimics, miR-455-3p mimics +vector, or miR-455-3p mimics +OE-Twist1 (*n* = 3). (The data are presented as the mean ± SD, ^∗^*P* < 0.05, ^∗∗^*P* < 0.01 versus the negative control).

To further investigate the target genes of miR-455-3p, we searched the potential mRNA targets through the miRanda and RNAhybrid databases and found that Twist1 expression was directly regulated by miR-455-3p. qRT–PCR results revealed that the Twist1 mRNA level was significantly lower in OEC than in YEC (Figure 4(a)). Twist1 mRNA was also downregulated after transfection of the miR-455-3p mimic in YEC (Figure 4(b)). A western blot analysis showed that the miR-455-3p mimic resulted in decreased Twist1 expression (Figure 4(c)). In the luciferase assay, miR-455-3p significantly inhibited luciferase activity in the MAECs transfected with Twist1 3'-UTR-WT, but not in the MAECs transfected with Twist1 3′-UTR-MUT (Figure 4(d)). Then, we transfected Twist1 siRNA into YEC to silence the Twist1 expression. A tube formation assay showed that Twist1 siRNA impaired the tube formation capacity of YEC (Figure 4(e)). In a Transwell assay, the migratory capacity of the YECs was also reduced after Twist1 silencing (Figure 4(f)).

Rescue experiments were performed to estimate the regulation of angiogenesis by miR-455-3p via Twist1 mRNA. The YEC proliferation rate was decreased after transfection of the miR-455-3p mimic, but this attenuation was prevented by Twist1 overexpression (Figure 4(g)). The *in vitro* wound healing assay results showed that upregulation of Twist1 expression reversed the low migratory capacity of YEC resulting from transfection of the miR-455-3p mimic (Figure 4(h)).

### 3.7. Twist1 Is a Transcription Factor that Regulates VEGFR2 Expression

**Figure 5:**
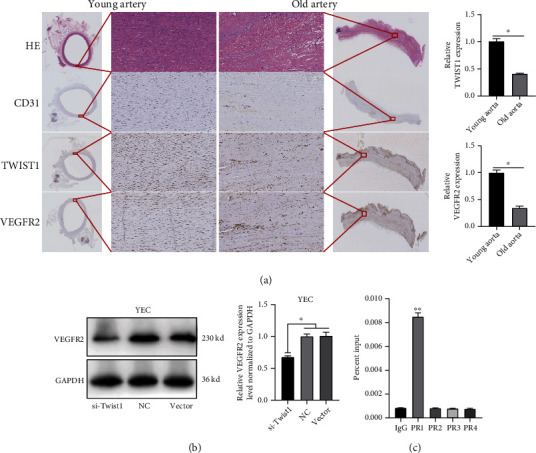
Twist1 acts as a transcription factor to regulate VEGFR2 expression. (a) Representative images of immunohistochemistry staining for Twist1 and VEGFR2 and relative expression levels of young patients' arterial and old patients' arterial tissue (*n* = 3). (b) Western blot analysis of the Twist1 expression in YECs transfected with si-Twist1 or NC (*n* = 3). (c) The CHIP experiment followed by qRT–PCR results showed that Twist1 was coprecipitated with the VEGFR2 primers 1. (The data are presented as the mean ± SD, ^∗^*P* < 0.05, ^∗∗^*P* < 0.01 versus the negative control).

Twist1 is a transcription factor that regulates gene expression by binding to the promoter region. We performed a bioinformatics prediction analysis using the Jaspar database. Notably, the VEGFR2 gene sequence was potentially targeted by Twist1 through four binding sites. To verify the age-related expression of Twist1, immunohistochemistry staining for Twist1 and VEGFR2 was performed and showed that, compared to that of old patients, the arterial tissue of young patients exhibited higher expression (Figure 5(a)). Furthermore, western blot analysis showed that the VEGFR2 protein level was significantly decreased in YEC transfected with Twist1 siRNA (Figure 5(b)). To further pinpoint the predicted binding sites between VEGFR2 and Twist1, four primers for the VEGFR2 gene promoter region were applied to a ChIP–qPCR experiment. The qRT–PCR results showed that Twist1 coprecipitated with primer 1. These results indicated the interaction between Twist1 and VEGFR2 gene promoter regions (Figure 5(c)).

### 3.8. CircCRIM1 Ameliorated Angiogenesis in Lower Limb Blood Flow Recovery in Aging Mice

We aimed to characterize the function of circCRIM1 in age-related lower limb ischemia *in vivo*. Aging mice were subjected to unilateral femoral artery ligation that cut off blood flow in the hind limbs, which was realized when the gastrocnemius muscle was injected with PBS or AAV9 particles carrying circCRIM1 or a vector 2 weeks before the establishment of the model, while the contralateral hind limbs and young mice injected with PBS on ipsilateral hind limbs were established as NC.

**Figure 6:**
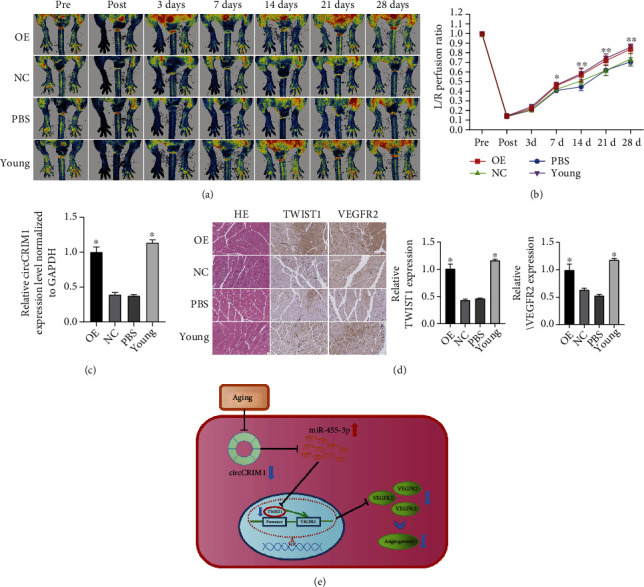
Overexpression of circCRIM1 ameliorated angiogenesis in lower limb blood flow recovery in aging mice. (a) Representative images of perfusion in the mice's femoral artery before and 0, 3, 7, 14, 21, and 28 days after the operation (*n* = 5). (b) Perfusion analysis was presented as the indexes by calculating the perfusion ratio of ischemic limbs to normal limbs before and 0, 3, 7, 14, 21, and 28 days after the operation (*n* = 5). (c) CircCRIM1 expression was markedly increased by AAV9-circCRIM1 in old mice gastrocnemius muscle sampled 2 weeks after the operation and was equivalent to that in the young group. (d) Representative images of the immunohistochemical staining for HE, anti-Twist1, and anti-VEGFR2 in mouse left lower limb gastrocnemius muscles sampled 28 days after surgery. The Twist1 and VEGFR2 expression were markedly increased in the circCRIM1 overexpression group compared with the NC group, and was equivalent to that in the young group (*n* = 5) (The data are presented as the mean ± SD, ^∗^*P* < 0.05, ^∗∗^*P* < 0.01 versus the negative control). (e) Schematic plot showed that circCRIM1 ameliorated angiogenesis in aging mice.

A PeriCam PSI imager was used to monitor blood flow before and 0, 3, 7, 14, 21, and 28 days after the operation (Figure 6(a)). The perfusion analysis showed that blood flow was increased 7, 14, 21, and 28 days after surgery in the circCRIM1 overexpression group compared with the NC group (Figure 6(b)), suggesting that circCRIM1 significantly improved blood flow recovery of the ischemic lower extremities *in vivo*. Furthermore, the qRT–PCR results confirmed that the abundance of circCRIM1 was markedly increased by AAV9-circCRIM1 in the gastrocnemius muscle sampled two weeks after the operation (Figure 6(c)). Consistent with these results, immunohistochemical staining for Twist1 and VEGFR2 in gastrocnemius muscle tissues in the circCRIM1 overexpression group sampled 28 days after surgery was also markedly increased compared with that in the NC group, and was equivalent to that in the young group. Taken together, these findings suggest that circCRIM1 acts as a miR-455-3p sponge inducing angiogenesis via the miR-455-3p/Twist1/VEGFR2 axis (Figure 6(e)).

## 4. Discussion

CircRNAs were initially discovered in virus research in the 1970s [10]. In the 1990s, an early report of circRNAs in eukaryotes showed that the mouse sperm-related gene Sry and the human ETS1 gene generated circRNAs through transcription [11, 12]. Previous studies showed that circRNAs were likely to be unfunctional byproducts of incorrect gene splicing due to a low abundance. However, with the rapid development of high-throughput sequencing in recent years, circRNAs have drawn a lot of interest with the characteristics of endogenous, abundant, conserved, and stable. CircRNAs participate in regulating the occurrence and development of various human diseases through transcriptional and protein translation [13, 14]. In this study, circCRIM1 was significantly decreased in OEC, and overexpression of circCRIM1 promoted OEC proliferation, migration, and tube formation capacity, suggesting that circCRIM1 was involved in EC angiogenesis in aging mice.

CircRNAs were revealed to action in the following ways: (1) as molecular sponges for miRNAs, adsorbing and sequestering miRNAs and thus playing a competitive inhibitory role; (2) as partners of RBPs, affecting RBP function; (3) as mediators of transcriptional regulatory elements by direct binding; and (4) as templates in protein-coding and translation [4, 15, 16].

Numerous studies support that circRNA can be miRNA sponges, which is an important aspect of the ceRNA regulatory mechanism hypothesis. It has been reported that ciRS-7 binds to AGO2 protein and competitively sponges miR-7, thereby regulating the expression of downstream target genes in the human brain [17]. CircRNA-Sry acts as a miR-138 sponge, thereby inhibiting its biological activity and playing a regulatory role in the occurrence and development of various pathologies [18]. Circ-ITCH acts in the miR-7/EGFR pathway to promote the migration and invasion of osteosarcoma cells through sponge adsorption [19]. CircHIPK3 elevates CCND2 expression and promotes cell proliferation and invasion in gliomas via miR-124 [20]. However, research on the ceRNA mechanism of circRNAs in aging ECs was infrequently reported.

Evidence of a high abundance in the cytoplasm and sufficient binding sites for target miRNAs proves that circRNAs can participate in regulating cellular functions by sponging miRNAs. In this study, we found that circCRIM1 was abundantly localized in the cytoplasm in mouse ECs through a FISH experiment. These results supported that circCRIM1 acts as a miRNA sponge. AGO2 is a core protein in RNA-induced silencing complexes (RISCs) that take charge of RNA interference and gene silence by cleaving target mRNA in the cytoplasm [21]. We found that circCRIM1 binds to the AGO2 protein Using RIP experiments, providing important evidence that circCRIM1 regulates the biological functions of vascular ECs through ceRNA mechanisms.

When seeking to identify the downstream target genes of circCRIM1, we found that miR-455-3p may be the target of circCRIM1 on the basis of the miRanda database and RNAhybrid database. To verify the interaction between circCRIM1 and miR-455-3p, qRT–PCR was performed after overexpressing circCRIM1 in OEC and knocking down circCRIM1 expression in YEC. The results showed that miR-455-3p was significantly and negatively correlated with the changes in circCRIM1 expression and that miR-455-3p was significantly more abundant in the OEC compared to YEC. Then, we performed FISH experiments and found that circCRIM1 and miR-455-3p colocalized in the cytoplasm. Moreover, a luciferase assay showed that miR-455-3p bound circCRIM1, thereby inhibiting circCRIM1 expression and regulating EC function in mice.

MiR-455-3p plays an inhibitory role in tumor angiogenesis, but its role in physiological angiogenesis affected by aging is still unclear. Therefore, after transfecting miR-455-3p mimics and inhibitors into MAECs, we found that inhibition of miR-455-3p expression enhanced OEC proliferation, tube formation, and migratory capacity, while YEC functions were attenuated after overexpression of miR-455-3p, as determined through a series of *in vitro* and *in vivo* experiments. In addition, we found stronger evidence of circCRIM1 regulating miR-455-3p through a rescue experiment. In OEC overexpressing circCRIM1, cotransfection of miR-455-3p mimics inhibited the angiogenesis-related phenotypes acquisition of OEC that had been enhanced by circCRIM1 upregulation. These results support that miR-455-3p is a downstream target of circCRIM1 and participates in regulating EC angiogenesis.

Studies have shown that miRNAs affected EC function by accelerating mRNA degradation and decreasing mRNA translation [22]. We performed bioinformatics analysis using the miRanda database and RNAhybrid database and found that Twist1 may be a downstream target gene of miR-455-3p. To further investigate Twist1 function, qRT–PCR was performed and showed that Twist1 mRNA level was significantly decreased in YEC transfected with the circCRIM1siRNA or miR-455-3p mimic, followed by western blotting determined that the Twist1 protein expression level was markedly downregulated. These results suggest that Twist1 expression was affected by miR-455-3p and circCRIM1. To further verify the relationship between Twist1 and miR-455-3p, we performed a luciferase assay and found that miR-455-3p bound Twist1 and inhibited Twist1 expression, but this phenomenon disappeared when the predicted binding site was mutated. Therefore, we confirmed that miR-455-3p is directly bound to Twist1 mRNA and regulated EC function in mice.

Studies have reported that Twist1 is a transcription factor that plays a role in cell development, tumor metastasis, and epithelial-mesenchymal transition in vertebrates [23, 24]. In various malignancies, such as sarcoma, melanoma, and glioblastoma, Twist1 is found that regulates tumor cell invasion and metastasis [25–27]. However, the study of the Twist1 in regulating angiogenesis has been controversial. The expression of Twist1 in the retinal capillary endothelium increased VEGFR2 expression, thereby promoting retinal angiogenesis in neonatal mice [28]. Nevertheless, the increased Twist1 expression with lower VEGFR2 levels was observed in older human adipocytes and mouse lung cells. And further overexpressing Twist1 in EC of mouse lung resection model was not related to the reduced VEGFR2 expression level and impaired proliferation or migratory capacity of EC. In contrast, overexpression of Twist1 increased VEGFR2 expression [29]. Intriguingly, we found that Twist1 was significantly downregulated in OEC compared with YEC. Therefore, we speculate that the role played by Twist1 in regulating vascular EC function may be tissue-specific.

To identify the role of Twist1 in MAECs, we found that the tube formation and migration rates were increased in the OEC overexpressing Twist1. Furthermore, the tube formation and migration rates were attenuated after the transfection of Twist1 siRNA in YEC. Then, we performed a rescue experiment and found that cotransfection of the Twist1 overexpressing plasmid released the inhibition of angiogenesis-related phenotype acquisition resulting from transfection of the miR-455-3p mimic in YEC.

Twist1 has been reported to be a transcription factor that binds the promoter region of VEGFR2 with an E-box domain that was highly conserved [28]. In this study, VEGFR2 expression was found to be parallel to Twist1 expression in MAECs. We predicted the possible binding sites of Twist1 to the promoter region of VEGFR2 on the basis of the Jaspar database. And then, based on the sense strand, we designed 4 pairs of primers for a ChIP–qPCR assay. The results showed that Twist1 bound to the promoter region of VEGFR2 (primer 1). We also determined the expression of Twist1 and VEGFR2 in arterial tissue in young and old patients through immunohistochemistry staining. And these results showed that the Twist1 and VEGFR2 expressions were significantly increased in young arteries than those in arteries. Therefore, it is believed that Twist1 promotes angiogenesis by promoting the expression of VEGFR2 in MAECs, which further supports the tissue-specific angiogenic effect of Twist1, as suggested in the literature.

To further study the effect of circCRIM1 on angiogenesis *in vivo*, we established a lower extremity femoral artery ischemia mice model and found that overexpressing circCRIM1 significantly accelerated the mouse ischemic limb blood flow recovery compared with those in the NC group and was equivalent to young mice group. The expression levels of Twist1 and VEGFR2 were higher in the OE-circCRIM1 group than in the NC group, as determined by immunohistochemistry staining. These results indicate that circCRIM1 can promote capillary regeneration after lower extremity ischemia and enhance blood flow recovery after acute lower extremity ischemia in aged mice *in vivo*.

## 5. Conclusion

In summary, we showed that upregulation of circCRIM1 reversed the angiogenic dysfunction of aging EC by targeting the miR-455-3p/Twist1/VEGFR2 axis *in vivo* and *in vitro*. This finding sheds light on a novel insight that circCRIM1 may be a potential therapeutic target for aging-related PAD.

## Data Availability

The data used to support the findings of this study are available from the corresponding author upon reasonable request.

## Supplementary Materials

Supplementary Materials Table I: SiRNA sequence for mouse gene used in this study. Table II: qRT–PCR primers for mouse genes used in this study. Table III: The primers for VEGFR2 used in ChIP–qPCR. Table IV: The probe sequences of circCRIM1 and miR-455-3p used in the FISH experiments.
