# Supplementary material for: CircCRIM1 Ameliorates Endothelial Cell Angiogenesis in Aging through the miR-455-3p/Twist1/VEGFR2 Signaling Axis
Source: Oxid Med Cell Longev. 2022 Oct 8;2022:2062885. doi: 10.1155/2022/2062885 (PMC9569221; doi:10.1155/2022/2062885)
Supplement: Supplementary Materials — Table I: SiRNA sequence for mouse gene used in this study. Table II: qRT–PCR primers for mouse genes used in this study. Table III: The primers for VEGFR2 used in ChIP–qPCR. Table IV: The probe sequences of circCRIM1 and miR-455-3p used in the FISH experiments. [file 2062885.f1.docx]

**Table I. SiRNA sequence for mouse gene**

| **Gene** | **Sequence 5’- 3’** |
| --- | --- |
| circCRIM1 siRNA-1 | GTGAAGATGAGGACTGGGA |
| circCRIM1 siRNA-2 | GTGTGAAGATGAGGACTGG |
| circCRIM1 siRNA-3 | TGTGAAGATGAGGACTGGG |
| miR-455-3p mimcs | GCAGUCCACGGGCAUAUACAC |
| miR-455-3p inhibitor | GUGUAUAUGCCCGUGGACUGC |
| Twist1 siRNA-1 | GGTACATCGACTTCCTGTA |
| Twist1 siRNA-2 | GACCTAGATGTCATTGTTT |
| Twist1 siRNA-3 | GCAAATAGATCCGGTGTCT |

**Table II. qRT**–**PCR primers for mouse genes**

| **Gene** | **Sequence 5’- 3’** |
| --- | --- |
| circCRIM1.F1 | CCTTCTGCCAGTGTATCAACG |
| circCRIM1.R1 | CATCCCAGTCCTCATCTTCACA |
| circCRIM1.F2 | TGTATCAACGGAGAACCTCACTG |
| circCRIM1.R2 | CATCCCAGTCCTCATCTTCACA |
| Twist1.F1 | CCGGAGACCTAGATGTCATTGT |
| Twist1.R1 | CCCACGCCCTGATTCTTGT |
| Twist1.F2 | TCTGAACACTCGTTTGTGTCCC |
| Twist1.R2 | CAGTGGCTGATTGGCAAGAC |
| U6.F | CTCGCTTCGGCAGCACA |
| U6.R | AACGCTTCACGAATTTGCGT |
| MiR-455-3p | RIBO-ssD9831042251 |

**Table III. Primers for VEGFR2 used in ChIP–qPCR**

| **Gene** | **Sequence 5’- 3’** |
| --- | --- |
| VEGFR2-F1 | TCTCTGTCTTCCACATGTCT |
| VEGFR2-R1 | TTGAACCTAGGACCAAGCAA |
| VEGFR2-F2 | CATACACAAAGTAAAGCAAGGTCT |
| VEGFR2-R2 | ATGAAATCTGGATATTTCTCCGAC |
| VEGFR2-F3 | TTTCATCCACCAGAGGTTTC |
| VEGFR2-R3 | ACTACAGGGAGAAGAGTCA |
| VEGFR2-F4 | AGATCCTGGAGCAGATGCAG |
| VEGFR2-R4 | GCTAGCACCAGGGATTTGCT |

**Table IV. The probe sequences of circCRIM1 and miR-455-3p in the FISH experiment**

| **Gene** | **Sequence 5’- 3’** |
| --- | --- |
| circCRIM1 | AUCAUCCCAGUCCUCAUCUUCACACACGGGGCAA |
| miR-455-3p | GTGTATATGCCCGTGGACTGC |
